# Night-time sleep duration and postpartum weight retention in primiparous women

**DOI:** 10.1093/sleepadvances/zpad056

**Published:** 2023-12-27

**Authors:** Jeanna T Ryan, Heather Day, Marlene J Egger, Jiqiang Wu, Christopher M Depner, Janet M Shaw

**Affiliations:** Department of Health and Kinesiology, University of Utah College of Health, Salt Lake City, UT, USA; Department of Family and Preventive Medicine, University of Utah School of Medicine, Salt Lake City, UT, USA; Division of Public Health, Department of Family and Preventive Medicine, University of Utah School of Medicine, Salt Lake City, UT, USA; Department of Family and Preventive Medicine, University of Utah School of Medicine, Salt Lake City, UT, USA; Department of Health and Kinesiology, University of Utah College of Health, Salt Lake City, UT, USA; Department of Health and Kinesiology, University of Utah College of Health, Salt Lake City, UT, USA

## Abstract

**Objectives:**

Approximately 75% of women weigh more at 1-year postpartum than pre-pregnancy. More than 47% retain >10 lbs at 1-year postpartum, which is associated with adverse health outcomes for mother and child. Disturbed sleep may contribute to risk of postpartum weight retention (PWR) as short sleep duration is associated with increased risk of obesity. Thus, we investigated whether night-time sleep duration is associated with risk for excessive PWR. We also explored night-time sleep duration and change in postpartum waist circumference.

**Methods:**

This is an ancillary analysis from a prospective cohort study. Participants were healthy primiparous adults with a singleton birth. Excessive PWR at 1-year postpartum was defined as ≥7% of pre-pregnancy weight. Log-binomial and linear regression assessed associations between night-time sleep duration at 6 months postpartum and PWR at 1-year postpartum. Linear regression assessed the association between night-time sleep duration and change in postpartum waist circumference.

**Results:**

Mean age of participants (*N* = 467) was 29.51 (SD ± 4.78) years. Night-time sleep duration by actigraphy or self-report was not associated with risk for excessive PWR (risk ratio 0.96, [95%CI 0.87–1.06]; risk ratio 0.95 [95%CI 0.83–1.07], respectively) or change in waist circumference.

**Conclusion:**

Night-time sleep duration at 6 months postpartum was not associated with PWR at 1-year postpartum. Mixed findings among our results and previous research could be due to our focus on night-time sleep, and differences in sleep measurement methods and timeframes across studies. More comprehensively assessing sleep, including multiple sleep dimensions, may help advance our understanding of potential links between sleep and PWR.

**Trial Registration:**

The parent study, Motherhood and Pelvic Health (MAP Study), is registered at https://clinicaltrials.gov/ct2/show/NCT02512016, NCT02512016.

**Statement of Significance**

Sleep during the postpartum period is commonly disturbed. Previous findings suggest a link between short sleep duration and postpartum weight retention. Our study, an ancillary analysis, investigated the relationship between night-time sleep duration at 6 months postpartum measured by actigraphy and self-report, and postpartum weight retention at 1-year as a dichotomous and continuous outcome. Neither night-time sleep duration by actigraphy nor self-report were associated with risk for postpartum weight retention. Differing sleep measurement methods and study designs, and our lack of daytime sleep measurements may contribute to mixed findings among prior studies and our current analyses. Remaining knowledge gaps exist regarding other aspects of sleep that may affect postpartum weight retention, especially in more generalizable and diverse populations.

## Introduction

Women endure complex social, physical, and psychological changes during the postpartum period, including changes in body weight and sleep [1–3]. Excessive postpartum weight retention (PWR) increases risk of adverse long-term health outcomes in mother and child including cardiometabolic risks and childhood obesity [4–6]. Furthermore, not achieving pre-pregnancy weight by 6 months postpartum increases risk for midlife obesity, diabetes, and heart disease [7]. Alarmingly, about 75% of women in the United States weigh more 1 year after childbirth than prior to pregnancy [7], with 1 in 5 women experiencing ≥5 kg PWR [8, 9]. Multiple factors are associated with PWR including pre-pregnancy body mass index (BMI), breastfeeding, parity, and employment status [10–12]. Gestational weight gain (GWG) appears to be the strongest predictor of PWR [6, 7, 10, 11, 13]. Poor sleep quality in late pregnancy is associated with excessive GWG, and women with excessive GWG and poor postpartum sleep quality are at risk of greater PWR [13–15]. These observations suggest sleep may be a potentially important factor moderating risk of PWR. Identifying additional modifiable factors of PWR, like sleep duration, could help identify new targets for effective interventions.

Variables such as newborn feeding may influence postpartum sleep [2, 16–18] and contribute to women commonly sleeping less than the recommended 7 hours/day [19] during the postpartum period. Notably, during the first 6 months postpartum versus late pregnancy, women have more wakefulness after sleep onset, decreased sleep efficiency, less nocturnal sleep, and less total sleep time [20, 21]. Such disrupted sleep is concerning because short sleep duration is associated with higher risk of obesity and larger waist circumferences in the general population [13, 14, 22, 23]. Moreover, short sleep duration (≤5 hours/day) in the postpartum period is associated with a higher waist circumferences at 3 years postpartum [24]. Previous research has demonstrated short sleep duration (≤5 hours/day) to be associated with PWR of ≥5 kg at 1-year postpartum [17], however, other researchers have found no association at 1-year postpartum [10]. Other findings have shown Black and Hispanic mothers with obesity who sleep <7 hours/night at 5 months postpartum have greater weight gain between 5 and 12 months postpartum compared to other participants [25]. Together, these data identify potential links between postpartum sleep and PWR.

Existing literature on postpartum sleep, however, is derived from inconsistent sleep measures and study designs, making direct comparisons across studies challenging. Research examining the relationship between actigraphy, self-reported sleep, and polysomnography (PSG) suggest low-to-no-correlation between methods in populations with disrupted sleep, including postpartum women [13, 26]. It is therefore plausible that sleep data from self-report versus wrist-actigraphy in postpartum women could produce different findings. Notably, Bland–Altman plots are a widely used and acceptable statistical method for analyzing agreement between measurements, like sleep duration, but its utilization in postpartum sleep assessment is scant [27–29]. To help address these gaps, our primary aim was to examine whether night-time sleep duration by wrist-worn actigraphy and self-report at 6 months postpartum is associated with risk for excessive PWR at 1-year postpartum. As an exploratory aim we describe the relationship between night-time sleep duration at 6 months and change in waist circumference from 5–10 weeks to 1-year postpartum. We hypothesized that shorter night-time sleep duration would be associated with excessive PWR and less change in waist circumference.

## Methods

### Participants and design

This is an ancillary study from the prospective cohort titled Motherhood and Pelvic Health (MAP), originally designed to examine pelvic floor health outcomes [30]. A full description of the parent study methods appears elsewhere [30]. At enrollment, participants were ≥18 years of age and in their third trimester (≥28 weeks gestation), nulliparous, English or Spanish speaking, ambulatory, and healthy with a singleton pregnancy. The parent study excluded participants if they did not have email or phone access, delivered before 37 weeks gestation, did not have a vaginal delivery, or were pregnant at or prior to 1-year postpartum. For the present analysis, we excluded participants without pre-pregnancy weight, valid actigraphy data and night-time wear, a response to self-reported sleep duration, or body weight at 1-year postpartum. Additionally, we excluded participants with missing data for GWG, breastfeeding, insurance, education, work status, and caffeine intake. The University of Utah Institutional Review Board approved all study procedures and methods. All participants provided written informed consent before initiating study procedures.

Demographic data, medical history, and employment status were collected by questionnaire or self-report at enrollment. Third trimester variables included age, ethnicity, race, education, health insurance, self-reported pre-pregnancy weight, and diagnosis of diabetes or hypertension. Caffeine and tobacco use were collected at 5–10 weeks postpartum. Actigraphy-derived light and moderate to vigorous physical activity was collected at 6 months postpartum. BMI, breastfeeding, and work status were collected at 1-year postpartum. Study staff measured weight (kg) by medical scale with participants wearing light clothing without shoes, height (centimeter (cm)) by stadiometer, and waist circumference with anthropometric tape at 5–10 weeks and 1-year postpartum.

### Night-time sleep duration measures

Self-reported sleep duration was assessed using question 4 (“during the past month . . . how many hours of actual sleep did you get at night?”) of the Pittsburgh Sleep Quality Index (PSQI) [31] administered within the 6 month window of MAP (5 months 0 days—7 months 30 days postpartum) [30]. Wrist-actigraphy was used to collect sleep and physical activity during the 6 month window of MAP [32]. Briefly, study staff instructed participants to wear a triaxial actigraph (Actigraph GT9X Link) continuously on their non-dominant wrist for seven consecutive days and nights. ActiLife 6.13.0 software was used to collect and manage data [33]. For inclusion, valid wear time was based upon acceptable standards for assessment of physical activity during waking hours at the time of study onset, and consisted of a minimum 10 hours of wear on four total weekdays plus at least 8 hours of wear on one weekend day [33–35]. Sleep derived from actigraphy was identified and processed using an R-package, GGIR V1.5-21, which is based on frequency of arm angle changes over 5-second epochs[36, 37]. Accuracy of this GGIR software without the use of sleep diaries found the mean *C*-statistic for detecting the sleep period time window compared to polysomnography was 0.83 in healthy sleepers [37]. A sustained inactivity bout (SIB) was defined as no change in arm angle by >5 degrees for >5 minutes [36]. Night-time sleep duration was calculated by totaling SIBs that occurred during the sleep period time window, 21:00 to 09:00 [30, 36].

### Weight and Waist Circumference

We calculated PWR by subtracting pre-pregnancy weight from weight at 1-year postpartum. A clinically defined threshold for PWR that poses significant health risks to mothers or their offspring is not defined, although a clinically meaningful weight increase of ≥3%–7% has been studied in other populations [38–42]. Thus, we conservatively used PWR of ≥7% and <7% to define excessive PWR. We also explored PWR as a continuous variable in case our 7% threshold was overly conservative.

We calculated GWG by subtracting pre-pregnancy weight from the highest weight assessed just before delivery, using the Institute of Medicine (IOM) guidelines to classify GWG as inadequate, adequate or excessive (Supplementary Table S1) [43, 44]. We determined waist circumference (cm) by averaging two measures taken at the natural waist with anthropometric tape at 5–10 weeks and 1-year postpartum [30]. Change in waist circumference was calculated as 1-year postpartum minus 5–10 weeks postpartum [30].

### Statistical analyses

Two-sample *t*-tests, Chi-square tests and Fisher’s exact tests compared demographic data between participants with ≥7% and <7% PWR. We described wear time and proportion of imputation for actigraphy with mean and SD.

We compared GWG and PWR at 1-year between participants with ≥7% and <7% PWR using two-sample *t*-tests and used mean, median, minimum, and maximum of PWR to describe participants by pre-pregnancy BMI and GWG categories. We compared mean night-time sleep duration from actigraphy and PSQI between participants with ≥7% and <7% PWR using two-sample *t*-tests. We used Bland–Altman analysis to analyze agreement between sleep duration from actigraphy and PSQI [45–47].

We described the mean and SD of participants’ waist circumference at 5–10 weeks postpartum and at 1-year postpartum. We described the mean and SD of waist circumference change from 5–10 weeks to 1-year postpartum by PWR.

To test our primary hypothesis, we conducted log-binomial regression models to calculate relative risk (RR) of excessive PWR at 1-year postpartum by night-time sleep duration at 6 months. Modified Poisson regression was used when log-binomial regression failed to converge. We conducted analyzed actigraphy and self-reported night-time sleep duration separately. We also conducted linear regression models using PWR as a continuous outcome. A directed acyclic graph (DAG; DAGitty version 3.0) guided selection of covariates for model adjustment [48, 49]. We reviewed prior evidence to support the causal unidirectional pathways among variables identified. We explored additional variables beyond the minimal sufficient adjustment set based on prior data, which were added to the minimal sufficient adjustment set model individually and separately.

Linear regression assessed night-time sleep duration and change in waist circumference from 5–10 weeks to 1-year postpartum.

Models were checked for sparsity, multicollinearity, and influential points. We determined minimal effect size and power given the data available. All statistical analyses were conducted using RStudio [50].

## Results

**Figure 1.**
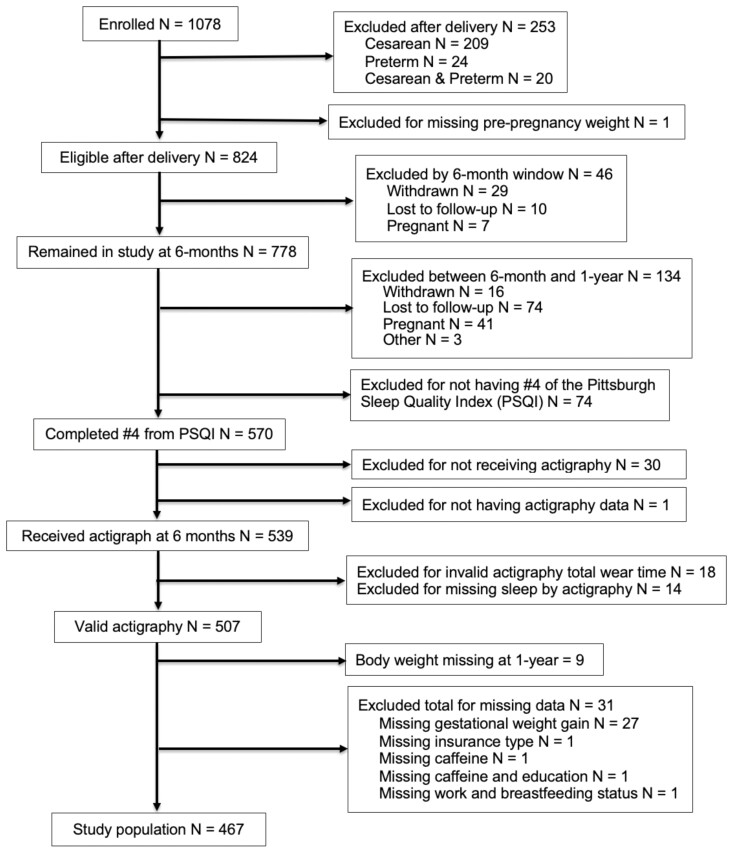
Participant flow chart showing the progression of participants starting at enrollment of the MAP study [30] through eligibility after delivery and exclusion criteria of this ancillary study.

A total of 76.4% (824/1078) of participants enrolled in the parent study were eligible after delivery (Figure 1), with 47% (507/1078) answering question 4 of the PSQI and having valid actigraphy data. After excluding 31 participants due to missing data, 43.3% (467/1078) remained eligible for the current analyses. The 357 participants who were missing pre-pregnancy or 1-year postpartum weight, withdrew, or had invalid actigraphy were more likely to be younger (26.56 ± 5.3 vs. 19.5 ± 4.8, *p* < 0.001), Hispanic (30.8% vs. 13.5%, *p* < 0.001), and have less education (*p* < 0.001) than those included, but were not significantly different in race or pre-pregnancy BMI. Table 1 shows baseline characteristics. The included participants were aged 29.51 ± 4.78 years with a pre-pregnancy BMI of 24.4 ± 5.03 kg/m^2^;more than 68% had a pre-pregnancy BMI < 25 kg/m^2^ [44]. More than 90% of participants were White and had at least some college education and health insurance. Power analysis showed our 467 analyzed participants provided 80% power with an alpha of 0.05 and 4 predictors to detect a minimal effect size of 0.03.

**Table 1.** Baseline participant characteristics and descriptive statistics for the total population of participants, and participants with ≥7% PWR and <7% PWR

Characteristic	Total population	≥7% PWR	<7% PWR	*p*
	(*N* = 467)	(*n* = 109)	(*n* = 358)	(*p < 0.05)
Age at enrollment, years				
(At delivery)				
Continuous, mean (SD)	29. 51 (4.78)	28.90 (5.41)	29.70 (4.57)	<0.124
Categorical, *n* (%):				*t*-test
18 to ≤25	126 (27.0%)	35 (32.1%)	91 (25.4%)	
26 to < 33	225 (48.2%)	47 (43.1%)	178 (49.7%)	0.343
≥33	116 (24.8%)	27 (24.8%)	89 (24.9%)	Chi-square test
Ethnicity, *n* (%)				
Non-Hispanic	404 (86.5%)	87 (79.8%)	317 (88.5%)	0.030*
Hispanic	63 (13.5%)	22 (20.2%)	41 (11.5%)	Chi-square test
Race, *n* (%) at screening				
White	426 (91.2%)	97 (89.0%)	329 (91.9%)	0.456
Non-white	41 (8.8%)	12 (11.0%)	29 (8.1%)	Chi-square test
Education, *n* (%)				
High school or less	31 (6.6%)	13 (11.9%)	18 (5.0%)	<0.001*
Some college or completed college	284 (60.8%)	75 (68.8%)	209 (58.4%)	Chi-square test
Graduate or professional degree	152 (32.5%)	21 (19.3%)	131 (36.6%)	
Health insurance type, *n* (%)				
Private/military	414 (88.7%)	86 (78.9%)	328 (91.6%)	0.001*
Medicaid/Medicare	34 (7.3%)	16 (14.7%)	18 (5.0%)	Fisher’s exact test
None	19 (4.1%)	7 (6.4%)	12 (3.4%)	
Pre-pregnancy body mass index (BMI) (kg/m^2^), mean (SD)	24.4 (5.03)	25.3 (5.34)	24.1 (4.91)	0.041*
Categorical, *n* (%):				*t*-test
BMI < 25	320 (68.5%)	71 (65.1%)	249 (69.6%)	
BMI 25 to <30	88 (18.8%)	19 (17.4%)	69 (19.3%)	0.226
BMI ≥ 30	59 (12.6%)	19 (17.4%)	40 (11.2%)	Chi-square test
Diabetes (total), *n* (%)				
Yes	23 (4.9%)	7 (6.4%)	16 (4.5%)	0.567
No	444 (95.1%)	102 (93.6%)	342 (95.5%)	Chi-square test
Hypertension (total), *n* (%)				
Yes	8 (1.7%)	2 (1.8%)	6 (1.7%)	1.00
No	459 (98.3%)	107 (98.2%)	352 (98.3%)	Fisher’s exact test
Gestational weight gain (kg)				
Continuous mean (SD)	14.20 (5.57)	16.89 (5.98)	13.38 (5.17)	<0.001*
Categories (*n*, %)				*t*-test
Inadequate	94 (20.0%)	10 (9.2%)	84 (23.5%)	
Adequate	148 (32.1%)	25 (22.9%)	123 (34.4%)	<0.001*
Excess	225 (48.0%)	74 (67.9%)	151 (42.2%)	Chi-square test

**Figure 2.**
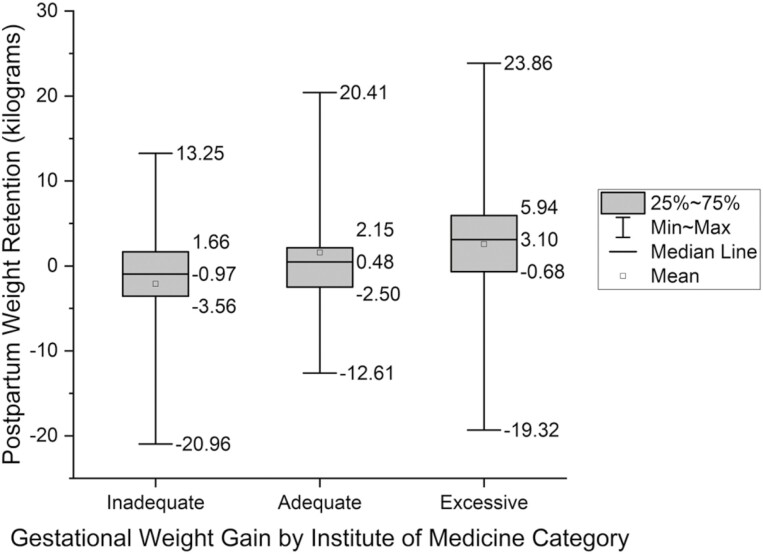
Postpartum weight retention by category of gestational weight gain (GWG). GWG was classified as inadequate, adequate or excessive based on category of prepregnancy body mass index (BMI) as defined by the Institute of Medicine. For underweight (BMI < 18.5 kg/m^2^), recommended GWG is 28–40 pounds (lbs). For normal weight (18.5–24.9 kg/m^2^), recommended GWG is 25–35 lbs. For overweight (BMI = 25–29.9 kg/m^2^), recommended GWG is 15–25 lbs. For obese (BMI≥30 kg/m^2)^, recommended GWG is 11–20 lbs (Supplementary Table S1). Inadequate GWG is less than recommended GWG for BMI, adequate is GWG within recommendations for GWG for BMI, and excessive is greater than recommended GWG for BMI [43].

**Figure 3.**
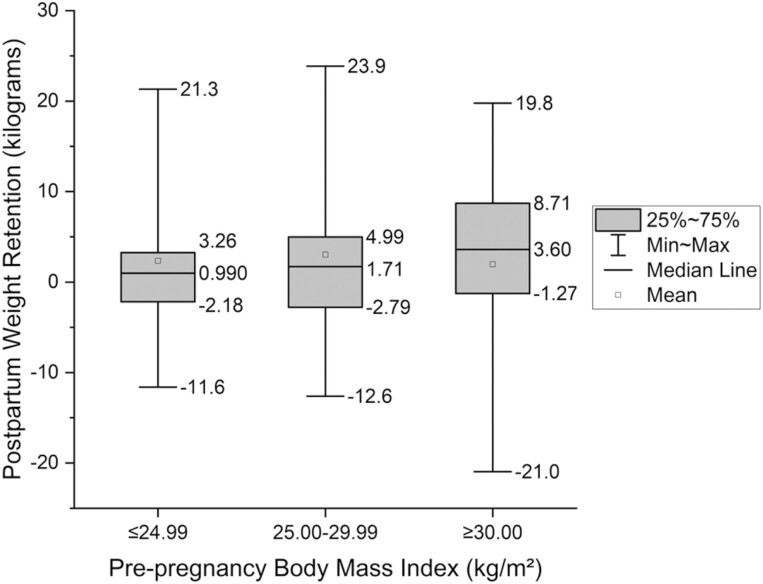
Postpartum weight retention by category of pre-pregnancy body mass index (kg/m^2^).

Table 2 shows participant characteristics from 5–10 weeks, 6 months, and 1-year postpartum. At 1-year postpartum, mean PWR was 1.5 kg (SD 5.8) and 23.3% (109/467) of participants had PWR ≥7%. Supplementary Figure S2 illustrates PWR for total participants, and participants with <7% and ≥7% PWR. Participants with ≥7% versus <7% PWR at 1-year postpartum were more likely to report Hispanic ethnicity (*p* = 0.03), have less education (*p* < 0.001), not be privately insured (*p* = 0.001), and less likely to be breastfeeding (*p* < 0.001) and working full-time (*p* = 0.01). There were no differences in age, race, caffeine, tobacco, diabetes, hypertension, or physical activity between groups. Additionally, participants with ≥7% PWR had 3.5 kg more GWG (*p* < 0.001) than participants with <7% PWR and more participants with ≥7% PWR had excessive GWG (*p* < 0.001). Participants with excessive GWG had more PWR (Figure 2, Table 1). Regarding BMI, participants with ≥7% PWR had a higher pre-pregnancy BMI and higher BMI at 1-year postpartum (*p* < 0.001) than those with <7% PWR (Figure 3, Table 1). Furthermore, the number of participants with a BMI in an overweight or obese category increased by 35% (38/109) at 1-year postpartum in the group with ≥7% compared to a decrease of 1% (5/358) in those with <7% PWR.

**Table 2.** Participant characteristics and descriptive statistics for the total population of participants, and participants with ≥7% PWR and <7% PWR at 5–10 weeks, 6 months, or 1-year postpartum

Characteristic	Total population	≥7% PWR	<7% PWR	*p*
	(*N* = 467)	(*n* = 109)	(*n* = 358)	(*p < 0.05)
Caffeine (yes/no) at 5–10 weeks *N* %				
No	148 (31.7%)	31 (28.4%)	117 (32.7%)	0.474
Yes	319 (68.3%)	78 (71.6%)	241 (67.3%)	Chi-square test
	Missing (*n*) = 0	Missing (*n*) = 0	Missing (*n*) = 0	
Tobacco (yes/no) at 5–10 weeks *N* %				
No (0)	460 (98.5%)	108 (99.1%)	352 (98.3%)	0.318
Yes (1)	5 (1.1%)	0 (0.0%)	5 (1.4%)	Fisher’s exact test
	Missing (*n*) = 2	Missing (*n*) = 1	Missing (*n*) = 1	
Light physical activity at 6 months (mean minutes/day ± SD)	352.63 (66.69)	349.64 (69.13)	353.54 (69.13)	0.593 *t*-test
	Missing (*n*) = 0	Missing (*n*) = 0	Missing (*n*) = 0	
Moderate to vigorous physical activity at 6 months (mean minutes/day ± SD)	87.79 (35.54)	83.27 (34.06)	89.16 (35.91)	0.130 *t*-test
	Missing (*n*) = 0	Missing (*n*) = 0	Missing (*n*) = 0	
Body mass index (BMI) (kg/m^2^) Mean (SD) at 1-year Categorical, *n* (%):	24.76 (5.62)	28.35 (6.14)	23.66 (4.96)	<0.001* *t*-test
BMI < 25	287 (61.5%)	33 (30.3%)	254 (71.0%)	
BMI 25 to <30	108 (23.1%)	45 (41.3%)	63 (17.6%)	< 0.001*
BMI ≥ 30	72 (15.4%)	31 (28.4%)	41 (11.5%)	Chi-square test
	Missing (*n*) = 0	Missing (*n*) = 0	Missing (*n*) = 0	
Currently breastfeeding, (yes/no) at 1-year *n* (%)				
No (0)	254 (54.4%)	78 (71.6%)	176 (49.2%)	<0.001*
Yes (1)	213 (45.6%)	31 (28.4%)	182 (50.8%)	Chi-square test
	Missing (*n*) = 0	Missing (*n*) = 0	Missing (*n*) = 0	
Work status at 1-year				
Working full-time (>30 hours/week)	248 (53.1%)	45 (41.3%)	203 (56.7%)	
Working part-time (<30 hours/week)	94 (20.1%)	25 (22.9%)	69 (19.3%)	0.014* Chi-square test
Other	125 (26.8%)	39 (35.8%)	86 (24.0%)	
	Missing (*n*) = 0	Missing (*n*) = 0	Missing (*n*) = 0	

PWR: postpartum weight retention.

**Figure 4.**
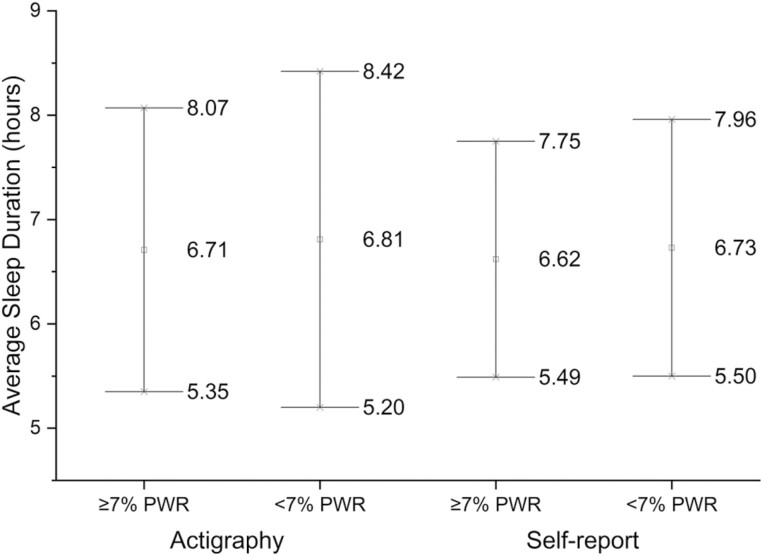
Mean ± SD (hours) of sleep duration by postpartum weight retention (PWR) of ≥7% versus <7% and sleep duration measurement method of actigraphy versus self-report.

Included participants had 7.1 ± 1.5 (SD) days and 22.9 ± 2.2 hours per day of valid actigraphy wear, with no difference between participants with <7% PWR versus ≥7% PWR (22.9 ± 2.3 hours versus 22.8 ± 2.1 hours, *p* = 0.79). Mean proportion of imputation was 4.8 ± 9.3%. Mean night-time sleep duration for all participants by actigraphy was 6.8 ± 1.6 hours and by self-report was 6.7 ± 1.2 hours. There was no difference in night-time sleep duration by actigraphy (*p* = 0.54) or self-report (*p* = 0.40) between participants with <7% PWR versus ≥7% PWR (Figure 4). The Bland–Altman plot showed no specific pattern of underestimating or overestimating between night-time sleep duration by actigraphy and self-report (Supplementary Figure S1). The bias, or mean of the differences between night-time sleep duration by actigraphy and self-report was 0.08 (95%CI −0.08, 0.24). The limits of agreement (LoA) were −3.41 (−3.69, −3.13) to 3.57 (3.29, 3.85) hours.

After adjusting variables per the DAG and confirming model convergence and no multicollinearity, we proceeded with log-binomial regression. The DAG determined a minimal sufficient adjustment set of pre-pregnancy BMI, GWG, and breastfeeding [48]. We adjusted for these variables in model 1. We adjusted for the minimal sufficient adjustment set plus physical activity, ethnicity, and health insurance in models 2, 3, and 4, respectively. Night-time sleep duration by actigraphy (RR 0.97, 95% CI 0.88–1.07, *p* = 057 and RR 0.96, 95%CI 0.87–1.06, *p* = 0.44) or self-report (RR 0.95, 95%CI 0.84–1.08, *p* = 0.42 and RR 0.95, 95%CI 0.83–1.07, *p* = 0.38) was not associated with risk of ≥7% PWR in the unadjusted model and the minimally adjusted model, respectively (Supplementary Table S2).

Adding the additional adjustment variables in models 2–4 did not meaningfully change this outcome (Supplementary Table S2). The RR for pre-pregnancy BMI was not statistically significant, but the RRs for excessive GWG and breastfeeding were statistically significant (Supplementary Table S4). The models in the linear regression analysis were also not significant for either measure of night-time sleep duration when PWR was treated as a continuous variable (Supplementary Table S3).

Mean waist circumference at 5–10 weeks postpartum was 81.8 ± 9.9 cm and at 1-year postpartum was 78.4 ± 11.4 cm. Mean change in waist circumference at 1-year postpartum from 5–10 weeks postpartum reflected a decrease by 3.5 cm (SD 5.2 cm). Participants with ≥7% PWR had 4.1 cm less mean change in waist circumference (*p* < 0.001) than participants with <7% PWR. Linear regression analyses showed change in waist circumference was not associated with night-time sleep duration by actigraphy (*β* −0.23, 95%CI −0.54 to 0.08, *p* = 0.15) or self-report (*β* 0.06, 95%CI −0.33 to 0.45, *p* = 0.77).

## Discussion

In this ancillary analysis of the MAP study, night-time sleep duration at 6 months postpartum, measured by actigraphy and self-report, was not associated with risk of ≥7% PWR at 1-year. Similarly, night-time sleep duration was not associated with PWR as a continuous outcome. Further adjusting the models for physical activity, ethnicity, or health insurance did not impact these overall findings. Moreover, night-time sleep duration by actigraphy and self-report were not associated with waist circumference change from 5–10 weeks to 1-year postpartum. Our findings also showed that GWG and breastfeeding had significant associations with excessive PWR, suggesting these factors are more strongly associated with PWR relative to night-time sleep duration measured at 6 months postpartum. Thus, contrary to some prior results, we did not find that night-time sleep duration was associated with risk of excessive PWR. However, because we did not assess daytime sleep or napping, which is common in this population, it is possible a more comprehensive assessment of sleep with more time points could produce different outcomes.

Similar to our results, Siega-Riz et al. found sleep duration ≤5 hours/day at 1-year postpartum was not associated with PWR at 1-year [10]. Dissimilar to our results, data from a prospective cohort study showed ≤5 hours sleep/day compared to 7 hours sleep/day at 6 months postpartum increased the odds of having ≥5 kg PWR at 1-year (odds ratio 3.13, 95% CI: 1.42, 6.94) [9]. Additional analyses from this longitudinal study also showed average sleep duration ≤5 hours/day, from 6 and 12 months postpartum, was associated with 1.5 kg (95% CI: 0.02, 2.86) more PWR at 3-years [24]. Results from another longitudinal study using a similar approach of averaging sleep duration from 6 weeks, 6 months, and 12 months postpartum [8], showed more sleep/day was associated with less PWR at 1-year (*B* −0.53, 95% CI: −1.08 to 0.02, *p* < 0.05) [8]. Given the poor sleep quality and variability in sleep duration across the postpartum period it is likely that one time point will not fully represent postpartum sleep [16]. Therefore, sleep duration at one point in time during the postpartum period may not provide enough sampling to reliably identify health outcomes linked with sleep duration. Findings show mother’s sleep is most disturbed during the first month after childbirth [16] with increased sleep opportunities by 6 months after childbirth, coinciding with infants ability to begin consolidating sleep by age 6 months [51–53]. This is why we assessed sleep at 6 months postpartum. Even so, there is high variability of sleep among infants, and sleep fragmentation of mothers may persist at 6 months postpartum [54]. As such, inconsistency in the literature on sleep duration and PWR may be due to the variability of when sleep duration measurement during the postpartum period occur, and whether sleep is defined by an average of multiple or a single time point(s).

Mixed findings on sleep duration and PWR may also be explained by differences in how studies assessed daytime sleep and napping. Previous findings show 26%–76% of women at 3–6 months postpartum report at least 1 nap/day [55–57]. We assessed sleep by actigraphy between 21:00 and 09:00, and asked for self-reported sleep that only occurred at night. The mean self-reported night-time sleep duration among our participants was 6.7 hours (SD 1.2), which is similar to other studies [8, 9, 13, 24]. Yet, most of the research mentioned above reported sleep that occurred over 24-hours [8, 9, 13, 24]. Matenchuk and Davenport matched sleep logs to actigraphy to identify napping, and found that participants with a PSQI ≥ 6 (“bad”) were more likely to report napping, leading to a higher total sleep duration [13]. We did not assess napping or daytime sleep [30], which could contribute to the mixed results between our study and others that assessed napping or 24 hours total sleep durations. Collecting sleep measurements over 24 hours, and by multiple methods concurrently could help avoid the limitations of comparing self-report versus actigraphy measured sleep dimensions across different studies.

Despite finding no association between ≥7% PWR at 1-year postpartum and night-time sleep duration at 6 months postpartum, there were differences in GWG and breastfeeding between participants with and without ≥7% PWR (Tables 1 and 2), which are consistent with previous research [6, 8–10, 13, 24, 25, 58]. High pre-pregnancy BMI, excessive GWG, and less time breastfeeding are established risk factors for PWR [7]. However, pre-pregnancy BMI > 30 kg/m^2^ compared to pre-pregnancy BMI < 25 kg/m^2^ was not associated with risk of having ≥7% PWR at 1-year in the present study. Although pre-pregnancy obesity was not associated with an increased risk of excessive PWR at 1-year postpartum in our population, pre-pregnancy obesity may be pertinent when assessing risk for postpartum weight gain differentiated from PWR [25]. Herring et al. examined sleep duration dichotomized as short sleep duration being <7 hours/night at 5 months postpartum with a continuous outcome of weight change between 5 and 12 months postpartum during the postpartum period [25]. They found Black and Hispanic mothers with obesity and short sleep duration gain more weight between 5 and 12 months postpartum (2.6 kg (Standard Error (SE) 0.8), *p* = 0.04) compared to other participants suggesting that postpartum weight gain may need to be considered its own adjustment variable [25].

Differences in adjustment variables between studies could be contributing to inconsistencies in the literature on sleep duration and PWR. Previous studies have adjusted for different combinations of variables that include GWG and breastfeeding as well as pre-pregnancy BMI, income, delivery mode, parity, depression, diet/nutrition, physical activity, age, race, ethnicity, education, employment status, marital status, television viewing, and sleep history [8–10, 13, 25]. Because of the complexity of interrelationships that are present amongst adjustment variables related to sleep duration and PWR, we used a DAG to identify a minimal sufficient adjustment set. Our results reinforce previous research and guidelines that target two of those adjustment variables, GWG and breastfeeding, in decreasing risk for excessive PWR [13, 43, 44, 58].

Night-time sleep duration at 6 months postpartum did not predict change in waist circumference from 5–10 weeks to 1-year postpartum in our population. Contrary to our findings, Taveras et al. found averaged self-reported postpartum sleep duration of ≤5 hours/day was associated with 3.1 cm higher waist circumference (95% CI: 1.27, 6.60) [24]. However, Taveras et al. examined waist circumference at 3-years postpartum rather than 1-year postpartum [24]. Additionally, our findings are exploratory because our first measure of waist circumference occurred at 5–10 weeks postpartum, which does not reflect a true baseline. Further research on the relationship between sleep duration and waist circumference in the postpartum period is needed before concluding its application in a clinical setting.

A key strength of our study is that we measured night-time sleep duration by actigraphy and self-report. Alternatively, the studies on postpartum sleep duration are largely based on either actigraphy or self-report [8–10, 24]. Although our findings showed no bias between actigraphy and self-reported night-time sleep duration, the LoA were wide (−3.41, 3.57 hours), suggesting actigraphy and self-report are not interchangeable methods in postpartum women. Notably, the PSQI queries sleep that occurred over the prior month whereas actigraphy was collected over an average of about one week. These different timeframes of data collection are one possible factor contributing to the wide LoA between actigraphy and sleep diaries. Plus, previous findings of MAP in a smaller group of participants showed over 90% of sleep assessed by PSQI preceded actigraphy assessment [35], which may have also contributed to the wide LoA. Our *apriori* defined outcomes were focused on sleep duration and not other dimensions of sleep such as regularity, satisfaction, alertness, timing, and efficiency [59, 60]. Prior work from a subset of MAP participants demonstrated that 59% of mothers had poor sleep quality, operationalized as a composite PSQI score of >5, at 6 months postpartum [35]. Given the high levels of sleep disruption and variability in postpartum women as well as the limitations of our study to focus exclusively on night-time sleep, future research specifically designed to uncover potential associations between the multiple dimensions of sleep health and PWR will help advance the field. Collecting sleep measurements over 24 hours, and by multiple methods concurrently could help avoid the limitations of comparing self-report versus actigraphy measured sleep dimensions across different studies. Importantly, to rigorously capture some dimensions of sleep health, notably regularity, modeling data show >7 days of actigraphy data are needed [61]. In populations with high variability, like postpartum women, larger sample sizes and up to 4 weeks of actigraphy data may be necessary to provide rigorous analyses [61].

Strengths of our study include the prospective design, a large population size with a statistical power of 80%, and night-time sleep duration data from actigraphy and self-report. However, the parent study was originally designed to investigate pelvic floor health outcomes [30]. Thus, we lacked information on potential confounders such as diet and depression. We also did not have sleep duration from before or during pregnancy, napping, sleep diaries, or factors that influence circadian rhythm such as light exposure. Street et al. studied OSA prevalence in pregnancy and resolution in postpartum, and found 37% of their participants were diagnosed with gestational sleep apnea, and 73% had not resolved by 6–8 months postpartum [62]. Therefore, a limitation of our study as well as previous studies was not accounting for sleep disorders such as OSA [8–10, 13, 25]. More research on sleep disorders in pregnancy and postpartum is an important future direction to advance the field.

Our study had other limitations. Like some previous research on postpartum sleep, the PSQI queries self-reported sleep duration only at night. However, the PSQI was not originally designed for use in the postpartum period when night-time sleep may underrepresent total daily sleep duration [13, 63, 64]. Breastfeeding status was collected at 1-year postpartum, which may have underestimated the number of participants breastfeeding at 6 months. Additionally, selecting a primiparous cohort automatically corrected for parity as a confounder, enhancing scientific rigor, but also limited generalizability to multiparous mothers [16]. Results may also lack generalizability to younger, less educated, and non-White populations.

Our study supports previous research substantiating that childbearing is a risk factor for overweight and obesity and demonstrates the need for novel interventions designed to mitigate excessive PWR in this population [7, 44]. Identifying whether sleep returns to a pre-pregnancy baseline, and understanding the intraindividual differences of sleep patterns and napping throughout the postpartum period and beyond could better inform sleep assessment and other health outcome predictors. Tools validated specifically for studying sleep during the postpartum period, and collecting multiple dimensions of sleep health are needed. Further, continued research on how sleep modifies factors like pre-pregnancy BMI, GWG, and breastfeeding could help guide interventions for lowering risk of overweight and obesity from childbearing. Sleep during the postpartum period is complex and difficult to study; despite these findings, it is important to investigate as behaviors during this period could impact future health.

## Data Availability

The data supporting this article is available from the corresponding author by reasonable request.

## Supplementary Material

zpad056_suppl_Supplementary_Tables_S1-S4_Figures_S1-S2
