# Supplementary material for: Night-time sleep duration and postpartum weight retention in primiparous women
Source: Sleep Adv. 2023 Dec 27;5(1):zpad056. doi: 10.1093/sleepadvances/zpad056 (PMC10838128; doi:10.1093/sleepadvances/zpad056)
Supplement: zpad056_suppl_Supplementary_Tables_S1-S4_Figures_S1-S2 [file zpad056_suppl_supplementary_tables_s1-s4_figures_s1-s2.docx]

Night-time Sleep Duration and Postpartum Weight Retention in Primiparous Women

Ryan JT, DAY H, EGGER MJ, Wu J, Depner CM, and Shaw JM

university of utah, College of health, Health & kinesiology

Corresponding author: Jeanna Tachiki Ryan

250 S 1850 E, Salt Lake City, UT 84112

jeanna.ryan@utah.edu

Jeanna T. Ryan, PhD Candidate, MPAS, PA-C, MSCIS, MS, RDN: Department of Health and Kinesiology, University of Utah College of Health, Salt Lake City, UT, USA.

Heather Day, MS: Department of Family and Preventive Medicine, University of Utah School of Medicine, Salt Lake City, UT, USA.

Marlene J. Egger, PhD, MS: Division of Public Health, Department of Family and Preventive Medicine, University of Utah School of Medicine, Salt Lake City, UT, USA.

Jiqiang Wu, MSc: Department of Family and Preventive Medicine, University of Utah School of Medicine, Salt Lake City, UT, USA.

Christopher M. Depner, PhD: Department of Health and Kinesiology, University of Utah College of Health, Salt Lake City, UT, USA.

Janet M. Shaw, PhD, FACSM: Department of Health and Kinesiology, University of Utah College of Health, Salt Lake City, UT, USA.

**Figure S1:** Bland-Altman Plot for Assessing Agreement between Sleep Duration by Actigraphy and Self-report. The Y-axis is the Differences of Sleep Duration (hours) by Actigraphy Minus Sleep Duration (hours) by Self-report. The X-axis is the Means for Sleep Duration (hours) by Actigraphy and Self-report.


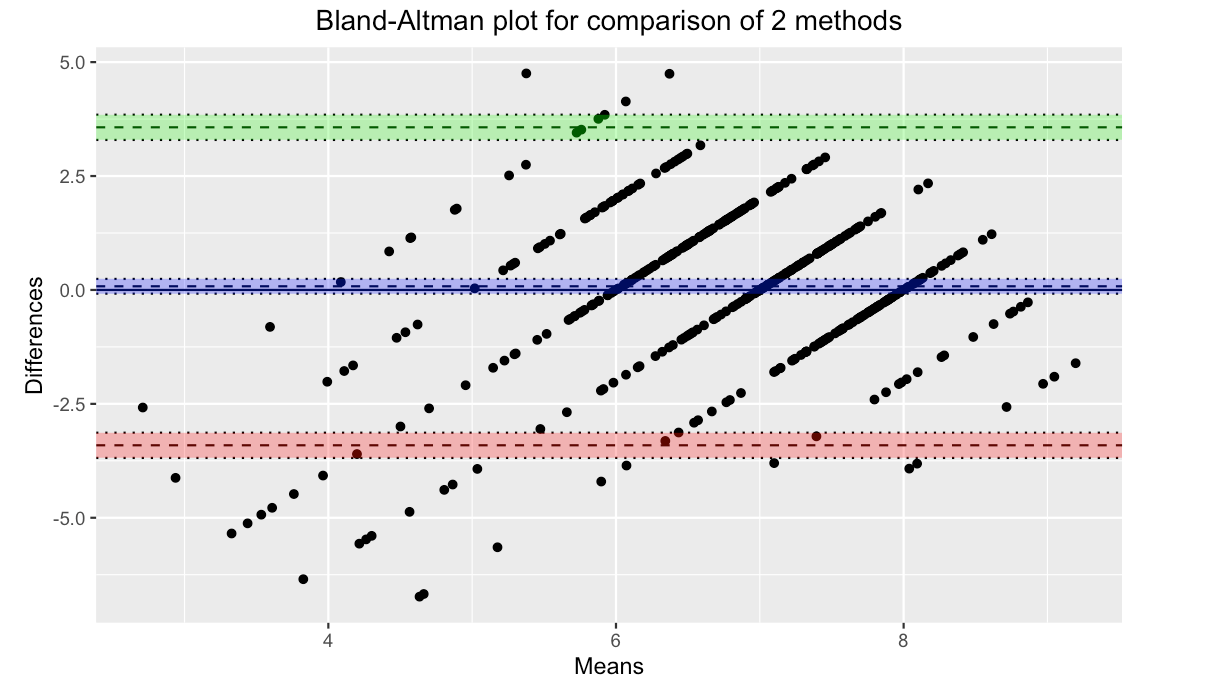


**Figure S2.** Postpartum weight retention (PWR) at 1-year for total population, participants with <7% PWR, and participants with ≥7% PWR.

**
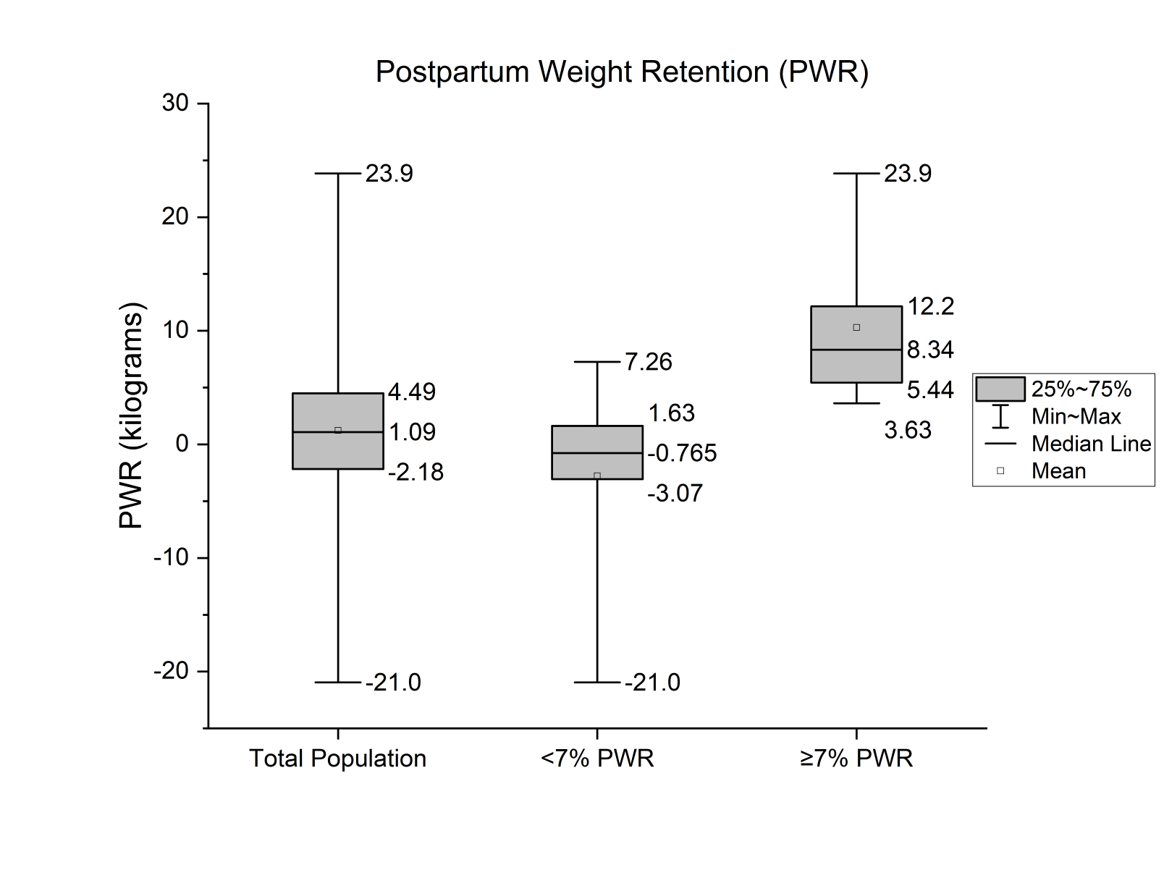
**

**Table S1.** Institute of Medicine’s (IOM) prepregnancy weight categories and recommended weight gain in pounds (lb) by body mass index (BMI) in (kilogram (kg)/meters (m)^2^).^1,2^

| **Category of prepregnancy BMI (kg/m^2^)** | **Recommendation for total weight gain (lb)** | **Classification as adequate, inadequate, or excessive weight change during pregnancy (lb)** |
| --- | --- | --- |
| Underweight (<18.5) | 28-40 | Adequate: between 28-40  Inadequate: <28  Excessive: >40 |
| Normal Weight (18.5-24.9) | 25-35 | Adequate: between 25-35  Inadequate: <25  Excessive: >35 |
| Overweight (25-29.9) | 15-25 | Adequate: between 15-25  Inadequate: <15  Excessive: >25 |
| Obese (>30) | 11-20 | Adequate: between 11-20  Inadequate: <11  Excessive: >20 |

**Table S2. Unadjusted and adjusted log-binomial regression models for sleep duration and postpartum weight retention.**

|  | Actigraphy | | | Self-report | | |
| --- | --- | --- | --- | --- | --- | --- |
| Model | Relative Risk (RR) | 95% Confidence Interval (CI) | p-value | RR | 95% CI | p-value |
| Unadjusted | 0.97 | 0.88, 1.07 | 0.57 | 0.95 | 0.84, 1.08 | 0.42 |
| Model 1^a^ | 0.96 | 0.87, 1.06 | 0.44 | 0.95 | 0.83, 1.07 | 0.38 |
| Model 2^b^ | 0.96 | 0.87, 1.05 | 0.37 | 0.96 | 0.85, 1.09 | 0.51 |
| Model 3^c^ | 0.97 | 0.88, 1.06 | 0.49 | 0.97 | 0.86, 1.09 | 0.61 |
| Model 4^d^ | 0.97 | 0.87, 1.06 | 0.48 | 0.95 | 0.84, 1.06 | 0.33 |

a. adjusted for pre-pregnancy body mass index (BMI), gestational weight gain (GWG) and breastfeeding

b. adjusted for pre-pregnancy BMI, GWG, breastfeeding and physical activity

c. adjusted for pre-pregnancy BMI, GWG, breastfeeding and ethnicity

d. adjusted for pre-pregnancy BMI, GWG, breastfeeding and health insurance; modified Poisson regression performed because Log-binomial regression failed to converge

**Table S3. Unadjusted and Adjusted Linear Regression Models for Sleep Duration and Postpartum Weight Retention.**

|  | Actigraphy | | | Self-report | | |
| --- | --- | --- | --- | --- | --- | --- |
| Model | Coefficient | 95% Confidence Interval (CI) | p-value | Coefficient | 95% CI | p-value |
| Unadjusted | -0.07 | -0.41, 0.27 | 0.69 | -0.08 | -0.52, -0.36 | 0.72 |
| Model 1^a^ | -0.14 | -0.46, 0.17 | 0.39 | -0.05 | -0.46, 0.36 | 0.81 |
| Model 2^b^ | -0.14 | -0.48, 0.19 | 0.40 | -0.05 | -0.46, 0.37 | 0.83 |
| Model 3^c^ | -0.10 | -0.42, 0.22 | 0.56 | -0.04 | -0.45, 0.36 | 0.83 |
| Model 4^d^ | -0.10 | -0.41, 0.22 | 0.55 | -0.06 | -0.46, 0.35 | 0.79 |

a. adjusted for pre-pregnancy body mass index (BMI), gestational weight gain (GWG) and breastfeeding

b. adjusted for pre-pregnancy BMI, GWG, breastfeeding and physical activity

c. adjusted for pre-pregnancy BMI, GWG, breastfeeding and ethnicity

d. adjusted for pre-pregnancy BMI, GWG, breastfeeding and health insurance

**Table S4. Log-binomial regression for minimal sufficient adjustment set variables and postpartum weight retention**

|  | Actigraphy | | | Self-report | | |
| --- | --- | --- | --- | --- | --- | --- |
| Variable | Relative Risk (RR) | 95% Confidence Interval (CI) | p-value | RR | 95% CI | p-value |
| >30 kg/m^2^ pre-pregnancy body mass index (BMI)^a^  Reference: <25 kg/m^2^ pre-pregnancy BMI | 1.13 | 0.76, 1.69 | 0.55 | 1.11 | 0.75, 1.67 | 0.60 |
| **Excessive gestational weight gain (kg)^a^**  Reference: adequate gestational weight gain | **1.90** | **1.26, 2.86** | **0.002** | **1.91** | **1.27, 2**.**88** | **0.002** |
| **Breastfeeding at 1-year postpartum^a^**  Reference: not breastfeeding at 1-year | **0.51** | **0.36, 0.74** | **<0.001** | **0.52** | **0.36, 0.74** | **<0.001** |

Bolded = statistically significant at <0.05, kg = kilogram, m = meter

a. Model 1: adjusted for pre-pregnancy body mass index (BMI), gestational weight gain (GWG) and breastfeeding

**References**

1. American College of O, Gynecologists. ACOG Committee opinion no. 548: weight gain during pregnancy. *Obstet Gynecol*. Jan 2013;121(1):210-2. doi:<http://10.1097/01.AOG.0000425668.87506.4c>

10.1097/01.aog.0000425668.87506.4c

2. Rasmussen KM YA, Institute of Medicine (US) and National Research Council (US) Committee to Reexamine IOM Pregnancy Weight Guidelines, eds. *Weight Gain During Pregnancy: Reexamining the Guidelines*. 2009. *The National Academies Collection: Reports funded by National Institutes of Health*.
